# Clusters of Rapid HIV Transmission Among Gay, Bisexual, and Other Men Who Have Sex with Men — United States, 2018–2021

**DOI:** 10.15585/mmwr.mm7138a1

**Published:** 2022-09-23

**Authors:** Stephen M. Perez, Nivedha Panneer, Anne Marie France, Neal Carnes, Kathryn G. Curran, Damian J. Denson, Alexandra M. Oster

**Affiliations:** 1 Division of HIV Prevention, National Center for HIV, Viral Hepatitis, STD, and TB Prevention, CDC.

Gay, bisexual, and other men who have sex with men (MSM) accounted for 68% of new HIV diagnoses in the United States in 2020* (*1*). Despite advances in treatment and prevention, HIV transmission among MSM continues, in part because of stigma and barriers to accessing prevention and treatment services (*2*). HIV cluster detection and response, a core strategy of the Ending the HIV Epidemic in the United States initiative,^†^ is an important tool for early identification and response to rapid HIV transmission, including among MSM. To better understand rapid HIV transmission among this population, CDC characterized large HIV molecular clusters detected using analysis of HIV-1 nucleotide sequence data from the National HIV Surveillance System (NHSS).^§^ Among 38 such clusters first detected during 2018–2019 that had grown to include more than 25 persons by December 2021, 29 occurred primarily among MSM. Clusters primarily among MSM occurred in all geographic regions, and 97% involved multiple states. Clusters were heterogeneous in age, gender identity, and race and ethnicity and had rapid growth rates (median = nine persons added per year). The overall transmission rate at cluster detection was 22 transmission events per 100 person-years, more than six times that of previously estimated national transmission rates (*3*). Most clusters of rapid HIV transmission occur among MSM. Swift response to reach diverse persons and communities with early, tailored, and focused interventions is essential to reducing HIV transmission (*4*).

Each calendar quarter, CDC analyzes HIV-1 polymerase (*pol*) sequences that are generated from routine HIV drug resistance testing as part of standard of care and reported to NHSS, to detect and notify jurisdictions of molecular clusters that are indicative of closely related transmission events and rapid transmission. Among persons with HIV infection diagnosed during the most recent 3 years, clusters are inferred using a pairwise threshold of 0.005 nucleotide substitutions per site; clusters of rapid transmission are those with five or more diagnoses during the most recent 12 months (*5*). Clusters first detected during 2018–2019 were examined, and large clusters were defined as those that had grown to include more than 25 persons as of December 2021. Each cluster was categorized according to the primary transmission category for persons in the cluster.^¶^ To better understand rapid transmission among MSM, further analysis was restricted to large clusters primarily involving MSM. Data reported through December 2021 were analyzed to describe these cluster characteristics and growth.

Demographic characteristics, transmission category,** and geographic information (U.S. Census Bureau region^††^ and CDC’s National Center for Health Statistics Urban-Rural Classification Scheme for Counties^§§^) were described for all persons and clusters. Annual growth rates were calculated as the increase in the number of persons in each cluster divided by the number of years between date of detection and December 2021. For clusters primarily comprising subtype B sequences,^¶¶^ previously established methods were used to estimate HIV transmission rates at the time of cluster detection*** (*5*); transmission rates were reported as the number of transmission events per 100 person-years. This activity was reviewed by CDC and was conducted consistent with applicable federal law and CDC policy.^†††^

Among 136 HIV molecular clusters with rapid transmission first detected during 2018–2019, 38 (28%) clusters exceeded 25 persons by December 2021; these 38 clusters accounted for 1,533 (53%) of all 2,901 persons in the 136 molecular clusters. At that time, 29 (76%) of the 38 clusters primarily involved MSM, six (16%) primarily involved persons who inject drugs, and three (8%) had no identified primary transmission category.

The 29 large clusters primarily among MSM included 985 persons, 52% of whom were aged 20–29 years at HIV diagnosis; 91% were male (Table 1). Thirty-four percent were Black or African American (Black) persons; 29% were Hispanic or Latino (Hispanic), and 29% were White. The most common transmission category was male-to-male sexual contact (MMSC) (77%); an additional 5% of persons reported MMSC and injection drug use.

**TABLE 1:** Characteristics of persons in large HIV clusters primarily among gay, bisexual, and other men who have sex with men (N = 29) — United States, 2021*

Characteristic	No. (%) of persons
**Total**	**985 (100.0)**
**Age group at HIV diagnosis, yrs**	
13–19	104 (10.6)
20–29	515 (52.3)
30–39	237 (24.1)
40–49	74 (7.5)
50–59	49 (5.0)
≥60	6 (0.6)
**Gender identity^†^**	
Male	898 (91.2)
Female	41 (4.2)
Transgender woman	41 (4.2)
Transgender man	4 (0.4)
Additional gender identity	1 (0.1)
**Race and ethnicity^§^**	
Black or African American	338 (34.3)
Hispanic or Latino	289 (29.3)
White	285 (28.9)
Multiracial	52 (5.3)
Asian	15 (1.5)
American Indian or Alaska Native	4 (0.4)
Native Hawaiian or other Pacific Islander	2 (0.2)
**Transmission category^¶^**	
Male-to-male sexual contact	759 (77.1)
Other or no identified risk	104 (10.6)
Heterosexual contact	49 (5.0)
Male-to-male sexual contact and injection drug use	44 (4.5)
Injection drug use	29 (2.9)
U.S. Census Bureau region**	
Northeast	149 (15.1)
Midwest	53 (5.4)
South	473 (48.0)
West	310 (31.5)
**Urbanicity^††^**	
Large central metro	516 (52.4)
Large fringe metro	178 (18.1)
Medium metro	193 (19.6)
Small metro	48 (4.9)
Micropolitan (nonmetro)	27 (2.7)
Noncore (nonmetro)	17 (1.7)
Missing urbanicity	6 (0.6)

* Includes molecular clusters first detected during 2018–2019 that included 25 or more persons as of December 2021 and for which more than 50% of persons were cisgender men (i.e., assigned male at birth and currently identify as male) who had a transmission category of male-to-male sexual contact.

^†^ Transgender woman includes persons assigned male sex at birth who identify as female. Transgender man includes persons assigned female sex at birth who identify as male. Additional gender identity includes persons assigned male or female at birth who do not identify as male, female, transgender woman, or transgender man and includes bigender, genderqueer, and two-spirit.

^§^ Hispanic or Latino persons can be of any race.

^¶^ Transmission category is classified based on a hierarchy of the risk factors most likely responsible for HIV transmission; classification is determined based on the person’s sex assigned at birth. Other risk factors include perinatal, hemophilia, and blood transfusion.

** https://www2.census.gov/geo/pdfs/maps-data/maps/reference/us_regdiv.pdf

^††^ https://www.cdc.gov/nchs/data/series/sr_02/sr02_166.pdf

Approximately one half (48%) of persons in these 29 clusters lived in the South U.S. Census Bureau region, followed by 31% in the West, and 15% in the Northeast; 5% lived in the Midwest. Overall, 70% of persons lived in large central metropolitan or large fringe metropolitan areas at the time of HIV infection diagnosis, and 20% lived in medium metropolitan areas.

As of December 2021, Black persons accounted for the largest racial or ethnic group in 13 (45%) large clusters among MSM, followed by White persons in nine (31%) clusters and Hispanic persons in seven (24%) (Table 2). In 14 (48%) clusters, the most common U.S. Census Bureau region was the South; 23 (79%) clusters included persons from more than one region. In 19 (66%) clusters, the most common area of residence was large central metropolitan; the second most common was medium metropolitan (seven clusters; 24%). Twenty-eight (97%) clusters involved persons in multiple states (median = four states; IQR = three to six states).

**TABLE 2:** Characteristics of large HIV clusters primarily among gay, bisexual, and other men who have sex with men,* by quarter — United States, 2018–2021

Quarter detected,^†^ cluster no.	No. of persons at detection	No. of persons as of Dec 2021	Annual growth rate^§^	Transmission rate at detection^¶^	As of Dec 2021 (% of persons in cluster)			
					Most prevalent age group at diagnosis, yrs	Largest racial and ethnic group**	Most common region^††,§§^	Most common urbanicity^††,¶¶^
**2018 Q1**								
1	11	30	5	20	30–39 (40)	Hispanic (50)	West (100)	Medium metro (70)
2	7	35	7	21	30–39 (31)	Hispanic (51)	South (86)	Large central metro (49)
3	9	27	5	—***	20–29 (52)	Black (63)	Northeast (93)	Large central metro (78)
**2018 Q2**								
4	6	27	6	36	20–29 (65)	Black (63)	West (74)	Large central metro (70)
5	9	40	9	50	20–29 40)	Black (35)	Northeast (88)	Large central metro (50)
**2018 Q3**								
6	17	56	12	25	20–29 (57)	White (88)	South (80)	Medium metro (54)
7	11	29	6	20	20–29 (53)	White (41)	South (79)	Large central metro (66)
**2018 Q4**								
8	12	38	9	26	20–29 (58)	Black (50)	South (71)	Large fringe metro (55)
9	14	32	6	39	20–29 (63)	White (75)	South (81)	Large central metro (75)
10	12	46	11	—***	20–29 (48)	White (35)	South (98)	Large central metro (74)
11	5	39	11	45	20–29 (51)	Hispanic (44)	Northeast (72)	Large central metro (49)
**2019 Q1**								
12	11	43	14	14	20–29 (44)	Hispanic (88)	West (100)	Large central metro (65)
13	11	33	10	23	20–29 (46)	Black (52)	Midwest (76)	Medium metro (48)
14	6	42	16	—***	20–29 (71)	Black (52)	West (90)	Large central metro (98)
15	9	34	11	22	20–29 (71)	Black (71)	South (94)	Large central metro (68)
16	6	30	11	43	20–29 (73)	Black (87)	South (100)	Medium metro (57)
17	15	36	9	23	13–19 (67)	Black (94)	South (94)	Large central metro (67)
18	9	27	8	—***	30–39 (41)	Hispanic (89)	South (100)	Large central metro (89)
19	15	26	5	21	20–29 (81)	Black (89)	South (100)	Small metro (62)
**2019 Q2**								
20	12	31	8	11	20–29 (77)	White (55)	West (94)	Large central metro (81)
21	8	29	8	15	30–39 (38)	Hispanic (86)	West (100)	Large central metro (97)
22	10	32	9	23	20–29 (47)	Hispanic (66)	West (97)	Medium metro (91)
23	16	26	4	23	20–29 (50)	Black (81)	South (96)	Large fringe metro (35) and medium metro (35)
**2019 Q3**								
24	5	37	14	140	30–39 (32)	White (81)	West (97)	Large central metro (51)
25	8	26	8	21	20–29 (54)	White (58)	Northeast (58)	Large central metro (62)
**2019 Q4**								
26	14	37	11	—***	20–29 (73)	Black (54)	West (86)	Large central metro (68)
27	9	44	17	—***	20–29 (43)	White (43)	South (95)	Large fringe metro (59)
28	11	27	8	25	20–29 (56)	Black (93)	South (81)	Large central metro (81)
29	19	26	3	21	20–29 (50)	White (69)	Northeast (96)	Medium metro (69)

**Abbreviations:** Q1 = quarter 1; Q2 = quarter 2; Q3 = quarter 3; Q4 = quarter 4.

* Includes molecular clusters first detected during 2018–2019 that included 25 or more persons as of December 2021 and for which more than 50% of persons were cisgender men (i.e., assigned male at birth and currently identify as male) who had a transmission category of male-to-male sexual contact.

^†^ Q1: January–March, Q2: April–June, Q3: July–September, Q4: October–December.

**^§^** Persons per year, calculated as the total number of cases added between the quarter of initial detection through December 2021, divided by the total years between initial detection and December 2021.

^¶^ Transmission events per 100 person-years estimated as the number of transmission events in a cluster, divided by the total time that persons in the cluster were living with HIV.

** Hispanic persons could be of any race.

^††^ Based on residence at time of diagnosis.

^§§^ https://www2.census.gov/geo/pdfs/maps-data/maps/reference/us_regdiv.pdf

^¶¶^ https://www.cdc.gov/nchs/data/series/sr_02/sr02_166.pdf

*** Subtypes are phylogenetically linked strains. Dashes indicate clusters composed primarily of persons with nonsubtype B sequences; transmission rates were not calculated for these clusters. HIV transmission rate methods use a subtype-specific substitution rate; these methods are only applied to the 23 clusters made up of subtype B sequences.

Median cluster size at the time of detection was 11 persons (IQR = 8–12); median size as of December 2021 was 32 persons (IQR = 27–38) (Figure). Median annual growth rate was nine persons per year (IQR = six to 11). Among 23 subtype B clusters, transmission rates ranged from 11 to 140 transmission events per 100 person-years (IQR = 21–31); the transmission rate across all subtype B clusters was 22 transmission events per 100 person-years.

**FIGURE:**
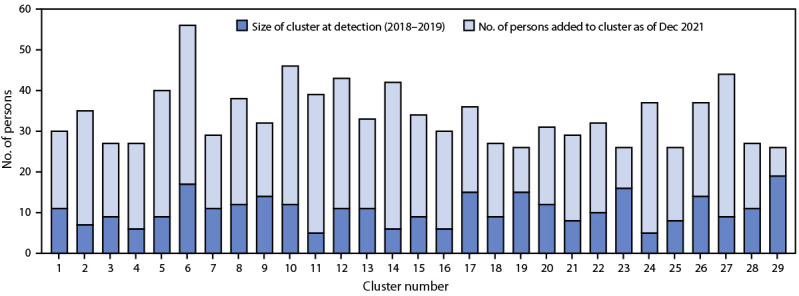
Increase in size of large HIV clusters primarily among gay, bisexual, and other men who have sex with men — United States, 2018–2021* * Clusters were detected during 2018–2019 and do not all have the same follow-up time from detection to December 2021.

## Discussion

This analysis found that most large clusters of rapid HIV transmission in the United States occur primarily among MSM. Such clusters were characterized by rapid growth and transmission rates more than six times those of previously estimated national rates (*3*).

The presence of an HIV cluster indicates a failure of treatment and prevention services to reach certain communities. HIV cluster detection and response activities can quickly identify rapid HIV transmission, including among MSM, and support early interventions that increase access to prevention and care services and improve health outcomes. These interventions should improve access and strengthen linkages to HIV testing, preexposure prophylaxis, and timely HIV treatment. Most clusters in this analysis were small at the time of detection, indicating an opportunity for these early interventions to uncover and address gaps in HIV services, remove any barriers to those services, and interrupt rapid transmission among MSM and others.

A recent analysis indicated that the characteristics of persons in HIV molecular clusters can vary geographically and over time, and that molecular analysis identifies rapid transmission that might not be evident from other surveillance data (*6*). In this analysis, the disproportionate representation of Black and Hispanic MSM in these clusters mirrored disparities observed in national HIV surveillance data *(1)*; however, the identification of clusters of rapid transmission provides a more local and nuanced understanding of diverse communities of MSM experiencing rapid transmission within larger heterogenous populations (e.g., all MSM). In addition to race and ethnicity, this analysis also identified variations in other characteristics of persons in large clusters primarily among MSM. While most persons in these clusters were cisgender men who reported MMSC, individual clusters also included transgender persons and persons who inject drugs. Health departments detecting and responding to these clusters can rapidly use data ascertained through cluster detection activities,^§§§^ as well as existing data sources (e.g., partner services data, other communicable disease surveillance data, and behavioral surveillance^¶¶¶^) or supplementary data collection (e.g., rapid needs assessments, qualitative interviews, and medical record abstraction), to better and more quickly understand affected populations and identify service gaps experienced by persons in these clusters (*4*,*7*). Gathering additional quantitative or qualitative data is important to understand and address the differing needs of persons in networks experiencing rapid transmission, including sexual, gender, and racial and ethnic minority groups involved in each cluster.

These cluster-specific data can guide the rapid implementation of response interventions (*4*). For example, clusters involving both Black and Hispanic MSM would benefit from interventions that address the unique needs and barriers faced by each group, rather than more generalized response activities aimed at broader MSM groups. Further, for clusters that primarily involve MSM but also include persons who inject drugs, response interventions should include activities to prevent both sexual and injection-related transmission. Persons involved in the clusters represented in this analysis vary in their prevention and treatment needs, barriers to accessing services, and experiences of stigma and discrimination (*4*,*7*); a single intervention is unlikely to be appropriate for all cluster responses, or for all persons within a cluster.

Clusters were detected in all regions of the country, and many included persons from multiple states, indicating the need for state and local health departments to be equipped to quickly detect and respond to clusters and collaborate with other health departments to address multistate clusters when indicated. CDC provides quarterly notification to jurisdictions about clusters of rapid transmission and supports health departments with guidance, tools, and technical assistance to implement cluster detection activities and build response programs**** that address the needs of MSM and others affected by HIV in their communities.

The findings in this report are subject to at least four limitations. First, incomplete HIV sequence reporting affects local and national cluster detection and characterization (*8*). Sequences were reported for approximately one half of diagnosed infections in recent years (*6*). Second, delays in sequence reporting can result in delayed cluster detection, artificially lowering estimates of growth rates for some clusters. Third, because sequences are available only for persons who have received an HIV diagnosis and entered care, persons in molecular clusters typically represent only a fraction of those in underlying transmission networks or in social networks who might have increased chances of acquiring HIV (*4*). Finally, this analysis does not include all clusters detected using other methods (*4*).

Most large, rapidly growing HIV clusters occur primarily among MSM. Leveraging cluster data to rapidly identify and implement interventions when clusters are first detected is essential to stopping transmission. Many MSM face barriers to accessing HIV services because of stigma, homophobia, racism, xenophobia, poverty, and limitations in health insurance^††††^ (*1*,*2*). Successful response interventions should aim to eliminate these barriers, quickly close service gaps, and address existing and emerging syndemics affecting MSM, including sexually transmitted infections and monkeypox (*9*). When mobilized effectively, strategies that engage communities, improve prevention services, and strengthen linkage to care can address the needs of persons in HIV clusters.^§§§§^ Understanding the diverse populations affected by HIV clusters among MSM is necessary to implementing tailored and robust response interventions, stopping transmission, and preventing new HIV infections in this population.

**Summary**

**What is already known about this topic?**

HIV molecular cluster detection and response activities identify communities in which rapid transmission is occurring and help guide public health action.

**What is added by this report?**

Most large HIV molecular clusters of rapid transmission occurred among gay, bisexual, and other men who have sex with men (MSM). These clusters occurred in all regions of the country, grew rapidly, and varied in demographic characteristics, including race and ethnicity.

**What are the implications for public health practice?**

Responding swiftly to clusters is important to interrupting transmission. Understanding the diverse populations in HIV clusters among MSM is necessary for implementing tailored and robust response interventions, improving prevention and care services, and stopping transmission in affected communities.

## Footnotes

* Includes infections attributed to male-to-male sexual contact only.

†
https://www.cdc.gov/hiv/library/reports/hiv-surveillance.html

§
https://www.hiv.gov/federal-response/ending-the-hiv-epidemic/key-strategies

¶ Clusters primarily among MSM are defined as those in which more than 50% of persons were cisgender men (i.e., assigned male gender at birth and currently identify as male) who had a transmission category of male-to-male sexual contact. Clusters primarily among persons who inject drugs are defined as those in which more than 50% of persons had a transmission category of injection drug use. Clusters with no primary transmission category are defined as those in which no single transmission category was common in more than 50% of persons in the cluster.

** Male-to-male sexual contact, injection drug use, male-to-male sexual contact and injection drug use, heterosexual contact, perinatal, or other. https://www.cdc.gov/hiv/library/reports/hiv-surveillance/vol-32/content/technical-notes.html

††
https://www2.census.gov/geo/pdfs/maps-data/maps/reference/us_regdiv.pdf

§§
https://www.cdc.gov/nchs/data/series/sr_02/sr02_166.pdf

¶¶ Subtypes, also called clades, are phylogenetically linked strains. HIV transmission rate methods rely on a subtype-specific substitution rate; these methods were only applied to the 23 clusters comprising subtype B sequences because subtype B is the most common HIV clade in the United States. The remaining six clusters primarily consisted of sequences from subtypes G and A, or sequences not able to be assigned a subtype.

*** Transmission rates at the time of cluster detection were estimated as the number of transmission events in a cluster, divided by the total time that persons in the cluster were living with HIV infection (i.e., the interval between the estimated date of infection and the date the cluster was detected in the analysis period). The number of transmission events in each cluster was calculated as the number of persons in the cluster minus one. To assess total time persons in the cluster were living with HIV infection, molecular clock phylogenetic analysis was used to estimate node ages or the time since transmission events connecting persons in the cluster.

††† 45 C.F.R. part 46, 21 C.F.R. part 56; 42 U.S.C. Sect. 241(d); 5 U.S.C. Sect. 552a; 44 U.S.C. Sect. 3501 et seq. Section 318(b-c) of the Public Health Service Act (42 USC Sect. 247c[b-c]), as amended, and the Consolidated Appropriation Act of 2016 (Pub. L. 114–113).

§§§ Jurisdictions are expected to conduct molecular analysis using their HIV surveillance data each month to detect HIV molecular clusters. These jurisdictions are supported in these analyses by programs made available by CDC and can detect, analyze, and visualize clusters using a bioinformatics tool called Secure HIV-TRACE. https://www.cdc.gov/hiv/funding/announcements/ps18-1802/guidance-relateds.html

¶¶¶
https://www.cdc.gov/hiv/statistics/systems/nhbs/index.html

****
https://www.cdc.gov/hivcluster

††††
https://www.cdc.gov/msmhealth/index.htm

§§§§
https://www.cdc.gov/hiv/policies/cdr/spotlights/texas.html
